# Epi4K: Gene discovery in 4,000 genomes

**DOI:** 10.1111/j.1528-1167.2012.03511.x

**Published:** 2012-05-29

**Authors:** The Epi4K Consortium

**Affiliations:** Center for Human Genome Variation, Duke UniversityDurham, North Carolina, U.S.A.

**Keywords:** Epilepsy, Epileptic encephalopathies, Genetics, Phenotyping, Prognosis, Sequencing, Copy number variants

## Abstract

A major challenge in epilepsy research is to unravel the complex genetic mechanisms underlying both common and rare forms of epilepsy, as well as the genetic determinants of response to treatment. To accelerate progress in this area, the National Institute of Neurological Disorders and Stroke (NINDS) recently offered funding for the creation of a “Center without Walls” to focus on the genetics of human epilepsy. This article describes Epi4K, the collaborative study supported through this grant mechanism and having the aim of analyzing the genomes of a minimum 4,000 subjects with highly selected and well-characterized epilepsy.

Great progress has been made in the past two decades in unraveling the genetic bases of epilepsy. Specific gene mutations, mostly involving ion channels and neurotransmitter receptors, are now recognized as the cause of rare inherited epilepsy syndromes. De novo mutations, including copy number variants (CNVs), are associated with some of the epileptic encephalopathies and a small percentage of patients with common epilepsy syndromes (Poduri & Lowenstein, 2011; Sisodiya & Mefford, 2011). However, the genetic mechanisms that influence the development of epilepsy in the vast majority of patients remain unknown. Recognizing the importance of accelerating progress in this area of epilepsy research, the National Institute of Neurological Disorders and Stroke (NINDS) issued a Funding Opportunity Announcement in 2010 for the creation of a “Center without Walls” to focus on the genetics of human epilepsy. This article describes Epi4K, the collaborative study supported through this mechanism and having the aim of sequencing (at least) 4,000 subjects with epilepsy. We begin with a general overview of epilepsy genetics, followed by a more detailed discussion of approaches and progress in gene identification in the complex epilepsies. We then describe the genesis of the Epi4K Consortium, including its structure and the development of policies meant to uphold key philosophical underpinnings of the nature of the collaboration. Finally, we provide details on the scientific rationale and proposed methods for the first four projects to be implemented by Epi4K.

## Overview of Epilepsy Genetics

The risk for epilepsy is clearly genetically influenced: risk is increased two- to fourfold in the first-degree relatives of people with epilepsy of unknown cause {either genetic (previously called idiopathic generalized epilepsies [IGEs]) or nonlesional focal epilepsies [NFEs]} (Annegers et al., 1982; Ottman et al., 1996a, b); twin studies consistently show higher concordance in monozygotic than dizygotic pairs (Corey et al., 1991; Berkovic et al., 1998; Kjeldsen et al., 2003; Vadlamudi et al., 2004); and a large number of genes with a major effect on susceptibility has already been identified (Helbig et al., 2008; Reid et al., 2009; Ottman et al., 2010). The study of rare families with Mendelian inheritance has provided extremely important information about epileptogenic mechanisms. The availability of large autosomal dominant pedigrees with epilepsy combined with the application of linkage analysis and positional cloning or positional candidate approaches has led to the identification of epilepsy genes that, to date, mostly encode subunits of voltage-gated or ligand-gated ion channels (Mulley et al., 2003; Prasad & Prasad, 2008; Poduri & Lowenstein, 2011). However, despite the clear importance of genetics in the epilepsies, most patients have no affected relatives, and progress in identifying and characterizing the underlying genetic defects in these “genetically complex” epilepsies has so far been slow (Mulley et al., 2005; Ottman, 2005).

Some of the genetic epilepsies are not inherited, but instead result from de novo mutations occurring in the germ cell line of the parents. Such de novo mutations are extremely important in severe myoclonic epilepsy of infancy (SMEI), now known as Dravet syndrome, in which >70% of patients have *SCN1A* mutations of which more than 90% arise de novo (Claes et al., 2001; Depienne et al., 2009). Cases with de novo mutations have also been found in genes initially identified in large families (Phillips et al., 2000; Bisulli et al., 2004; Claes et al., 2004; Bertrand et al., 2005; Michelucci et al., 2007; Ishii et al., 2009).

Recently, copy number variants (CNVs) have been found to play an important role in the genetically complex epilepsies, and many of these occur de novo (Scheffer & Berkovic, 2010). CNVs are deletions, duplications, or insertions of DNA that range in size from ∼1 kb to several megabases and may affect one or more genes. Three large, recurrent microdeletions at 15q13.3, 16p13.11, and 15q11.2 are each present in 0.5–1% of patients with epilepsy (Dibbens et al., 2009; Helbig et al., 2009; de Kovel et al., 2010; Mefford et al., 2010). Microdeletion of 15q13.3 appears to confer risk specifically for IGEs with an odds ratio of 68 (Dibbens et al., 2009). It is important to note that all three of these deletions are also established risk factors for related disorders, including intellectual disability, autism, and schizophrenia (Mefford & Mulley, 2010). Rare, nonrecurrent CNVs are also important. Large (>2 Mb), rare CNVs, including the deletion at 16p13.11, were found to be enriched in patients with diverse epilepsy syndromes (Heinzen et al., 2010). Rare CNVs were also found in ∼10% of patients with various types of epilepsy (Mefford et al., 2010) and ∼8% of patients with epileptic encephalopathies (Mefford et al., 2011).

## Gene Identification in the Complex Epilepsies

Historically, studies aimed at gene identification in complex disorders have used linkage and association approaches. Linkage studies rely on genetic and phenotypic information from multiple generations to map regions of the genome that are coinherited with disease in families. Regions of interest from linkage studies tend to be relatively large due to the few opportunities for recombination within the families investigated. Association studies, on the other hand, look for variants at individual loci that occur more commonly in unrelated patients than controls. Since association studies draw from many different unrelated individuals who each have a unique recombination history, the size of the associated region should theoretically be much smaller, and the identification of specific susceptibility genes may be more easily resolved than with a linkage study (Cardon & Bell, 2001). To identify the specific susceptibility genes generating a linkage signal, linkage studies are often followed by association mapping. This approach successfully identified the well-known apolipoprotein E (*APOE*) association with Alzheimer's disease (Pericak-Vance et al., 1991; Corder et al., 1993; Saunders et al., 1993), and the association of human leukocyte antigen (HLA) with many immune system disorders (Tomlinson & Bodmer, 1995).

Candidate gene association studies focus on genes thought likely to be involved in the disease pathophysiology. In epilepsy, the largest such study looked for associations with common variants in 229 candidate genes in 2,717 patients with epilepsy and 1,118 controls, and failed to identify any clear associations (Cavalleri et al., 2007b). Numerous small scale association studies in epilepsy have been published without yielding reproducible findings (Tan et al., 2010). Moreover, in a search for rare variants in idiopathic epilepsies, Klassen et al. (2011) resequenced protein coding regions of ion channel genes and did not find an excess of variation compared to controls. However, the small samples sizes may have precluded the identification of genetic risk factors of considerable effect sizes.

The lack of success of candidate gene studies in epilepsy and other complex diseases has been thought to result in part from inadequate knowledge of disease pathophysiology needed to select candidate genes. Consequently, the use of genome-wide association studies (GWAS), which allow for unbiased genome-wide association analyses, marked an important turning point in the field. GWAS employ genotyping chips to genotype “tagging” variants that capture almost all common variants (population frequency >5%) in the European and Asian populations, with slightly lower coverage for subjects of African ancestry (Barrett & Cardon, 2006). Prior to GWAS, only a handful of secure associations had been identified with linkage and association-based approaches in complex diseases (e.g., *APOE4*: Corder et al., 1993; Pericak-Vance et al., 1991; Saunders et al., 1993; HLA: Tomlinson & Bodmer, 1995); however, from 2005, when the first GWAS was published, to the middle of 2011, more than 1,449 associations have been published in more than 237 different conditions (Hindorff et al., n.d.). While some of these may be false positive, the majority of them satisfy significance thresholds sufficient to confirm the presence of real effects in the relevant genomic regions.

While GWAS have been successful in identifying secure risk factors for common diseases; the impact of these findings has been somewhat constrained by two principal factors: (1) the effects of the variants have been generally modest and, even cumulatively, often explain relatively small proportions of the presumed heritability; and (2) causal variants that underlie the association signals have proven difficult to identify, limiting the biological inferences that can be drawn (Goldstein, 2009; Manolio et al., 2009). In epilepsy only two GWAS have been published so far. The first, which included nearly 4,000 European patients with a wide array of nonlesional and lesional focal epilepsies, identified no secure associations (Kasperaviciute et al., 2010). Power was sufficient to exclude variants with an odds ratio of 1.3 or greater, and thus the results argue against common genetic effects of this magnitude that are shared across different (and likely heterogeneous) forms of focal epilepsy in European populations. In a GWAS of 1,087 focal epilepsy patients of Han Chinese decent, two suggestive but not definitive associations were reported, including one within a gene encoding a cytoskeletal protein (Guo et al., 2012). Analyses of smaller, more clinically homogenous subgroups (e.g., nonlesional focal epilepsies), in which family studies show a stronger genetic influence (Ottman et al., 1996a) may reveal associations masked by the clinical heterogeneity in both of these studies. GWAS are underway in other forms of epilepsy, including IGE, and meta-analyses using the data from a number of consortia (e.g., EPICURE, 2007; EPIGEN, 2008; Guo, 2012; Lohoff et al., 2005) are being undertaken through the efforts of a recently formed International League Against Epilepsy (ILAE) Consortium on Genetics of Complex Epilepsies. The success in identifying common variants that influence epilepsy susceptibility will rely on these collaborative efforts.

Given that GWAS have failed to identify many variants of large effect in complex disease, there is growing interest in the possibility that variants too rare to be represented well on GWAS chips may be important contributors to common diseases, including the epilepsies. This possibility has led to considerable interest in the use of next-generation sequencing (NGS), which allows for the near-comprehensive characterization of genetic variants across the genome, to identify rare risk factors for disease (Cirulli & Goldstein, 2010). The rare variant hypothesis is supported by the recent observation that rare CNVs can have large effects on neuropsychiatric disease risk (Need & Goldstein, 2010). This observation, in conjunction with the fact that only 9% of variants reported as disease-associated in the Human Gene Mutation Database are copy number variants (HGMD Professional version 2011.3) (Stenson et al., 2009), strongly suggests that other types of rare variants, such as single nucleotide and indel (insertion and/or deletions) variants, may be important contributors to complex disease. Early successes of next-generation sequencing in identifying the cause of several Mendelian diseases makes clear that pathogenic mutations can be found using this approach (Ng et al., 2010a, b; Sobreira et al., 2010), including recent studies in epilepsies (Corbett et al., 2010, 2011; Veeramah et al., 2012).

There is great potential for NGS to identify susceptibility genes for complex disease; however, two lines of evidence indicate that large samples sizes will be required. First, based on previous studies of epilepsy genetics, both locus and allelic heterogeneity are predicted to be high. Second, all genomes carry a large number of rare functional variants, which means that appropriately correcting for all the tested hypotheses requires very low p-values (Pelak et al., 2010). As discussed previously, direct evidence for the complexity of this variation in epilepsy was provided in a recent study that characterized the gene sequences of 237 ion channel genes in a cohort of individuals with IGE (Klassen et al., 2011).

Given the complexity of epilepsy genetics and the difficulty for any single group to have sufficient samples sizes for further gene discovery, the critical need for large-scale collaborative efforts has become increasingly clear.

## The Genesis of Epi4K

In late summer 2010, NINDS sponsored an international workshop on the genetics of human epilepsy in San Diego, attended by epilepsy genetics researchers from throughout the world, as well as leaders in genome sequencing, statistical genetics, and functional genomics. The goals of the meeting were to advance epilepsy genetics research by reviewing the state of current investigations, promoting collaboration, and brainstorming about new strategies likely to be successful in identifying and characterizing genetic effects on complex epilepsies.

**Figure 1:**
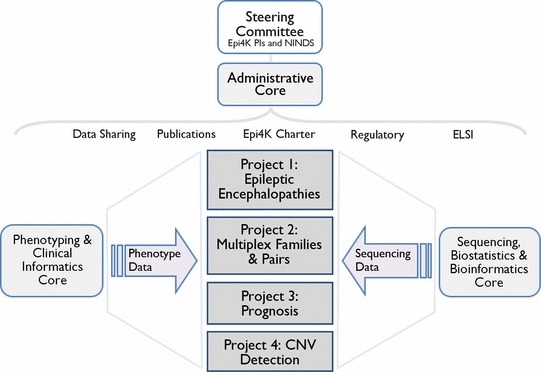
Epi4K organizational structure. See text for details. The terms directly under the Administrative Core refer to subcommittees charged with overseeing specific policies, procedures, and other issues relevant to collaborative genetic studies. ELSI, Ethical, Legal and Social Issues.

Following the workshop and internal discussions, NINDS issued a Funding Opportunity Announcement (FOA) in November 2010 to solicit applications for new “Centers without Walls for Collaborative Research in the Epilepsies: Genetics and Genomics of Human Epilepsies” (DHHS, 2010). The FOA encouraged applications from large, multidisciplinary groups and called for linked applications that could include an administrative core, a genomics core, and specific scientific projects. Catalyzed by this grant opportunity, we began meeting to identify the scientific questions of highest priority that could take advantage of the valuable epilepsy patient cohorts that have been assembled and the increased ability to apply NGS to the analysis of thousands of genomes. Our discussions also focused on the optimal structure for a Center without Walls, as well as our shared philosophy about key issues relating to collaboration and publication in multidisciplinary research. As a result, we developed a structure of three cores and four scientific projects, as well as a steering committee comprising the study primary investigators and representatives from NINDS (Fig. 1). The three cores include the Administrative Core (responsible for overall coordination of the Center's activities), the Sequencing, Biostatistics and Bioinformatics Core (responsible for all NGS and initial identification of sequence variants), and the Phenotyping and Clinical Informatics Core (responsible for verifying and archiving the phenotypic data associated with every DNA sample that will be sequenced). The projects, described in more detail in the following sections, include three that analyze specific sets of epilepsy cohorts (epileptic encephalopathies, multiplex families and pairs, and prognosis) drawn from seven large-scale genetic studies from throughout the world (Table 1), and one that will apply cutting-edge analytic techniques related to the detection of CNVs. The proposed number of patients to be analyzed in the scientific projects is a minimum of 4,000; thus we named the Center “Epi4K: Gene Discovery in 4,000 Genomes.” The planned timeline for this effort spans 5 years (beginning October 2011), and includes milestones for the completion of project 1 (epileptic encephalopathies) by the end of year 3 and project 2 (multiplex families and pairs) by the end of year 5. Completion of project 3 (prognosis) will depend on extra funds being obtained.

**Table 1:** Epi4K patient cohorts

Cohort	Patient types (numbers)	References
The Epilepsy Phenome/Genome Project (EPGP)	First-degree relative pairs with IGE or NFE (n = 1,500) Trios with IS (n = 250), LGS (n = 250) or malformations of cortical development (n = 250)	EPGP (2007)
Epilepsy Family Study of Columbia University	Multiplex families with three or more affected patients with IGE or NFE (n = 32)	Ottman et al. (2005); Ottman and Susser (1992)
The Epilepsy Research Centre, University of Melbourne	Multiplex families with three or more affected patients with IGE or NFE (n = 250)	Marini et al. (2004)
Quebec Familial Epilepsy Study	Multiplex families with three or more affected patients with IGE or NFE (n = 47)	Kinirons et al. (2008)
Childhood Absence Epilepsy Study (CAE)	Singletons with CAE (n = 446)	Glauser et al. (2010)
SANAD and University of Melbourne	Newly diagnosed singletons with 716 IGE (n = 716) or focal epilepsy (n = 1,721) prospectively followed for seizure outcome	Bleck (2007); Marson et al. (2007)
EPIGEN	Singletons with IGE (n = 700) or NFE (n > 3,000)	EPIGEN (2008)

IGE, idiopathic generalized epilepsy; NFE, nonlesional focal epilepsy; IS, infantile spasms; LGS, Lennox-Gastaut syndrome; CAE, childhood absence epilepsy.

To establish a clear, upfront understanding among all Epi4K investigators regarding the nature of the collaborative effort, an “Epi4K Charter” was drafted and signed by all investigators. The Charter, which can be found at http://www.epgp.org/epi4/charter, specifies guiding principles (including the concept of “no surprises”; i.e., that all members of Epi4K are required to present and discuss any plans that utilize Epi4K data or resources with the Epi4K Steering Committee), the organizational structure, and policies for decision making and data sharing. We also wrote an explicit Publication Policy (see http://www.epgp.org/epi4/PubPolicy) that provides detailed guidelines for the planning and generation of all reports that rely on information that has resulted from the collaborative nature of Epi4K. Among other things, the Publication Policy describes the primary papers expected to result from the Epi4K projects, and it stipulates that authorship for all primary papers will be under the title “The Epi4K Consortium.” The policy also emphasizes that a goal of Epi4K is to facilitate the career development of early-stage and midstage scientists and, to that end, priority will be given to junior investigators (defined as individuals who have not yet achieved the rank of full professor or its equivalent, including senior level postdoctoral fellows) when authors are named on secondary and other papers. Furthermore, senior investigators (those at the rank of full professor or equivalent) will, in general, not be named authors on Epi4K papers unless they have had a substantive role in being individually responsible for the conceptualization or analysis of the work.

## Epi4K Project 1: Epileptic Encephalopathies

Project 1 of the Epi4K Center Without Walls will address the genetics of epileptic encephalopathies, conditions defined by severe epilepsy that are typically refractory to medication accompanied by comorbid cognitive and behavioral dysfunction. While epileptic encephalopathies are extremely diverse, the study focuses on two of the more common types: infantile spasms (IS) and Lennox-Gastaut syndrome (LGS).

Infantile spasms are among the most common of the epileptic encephalopathies with an incidence of 1 in 3,000 live births and an onset between 4 and 12 months of life (Trevathan et al., 1999; Frost & Hrachovy, 2003). IS are associated with the characteristic chaotic interictal EEG pattern of hypsarrhythmia, the sine qua non of the syndrome, and the spasms themselves are associated with an electrodecremental response. About 50–65% of IS cases have known causes such as developmental brain malformations, tuberous sclerosis complex, chromosomal syndromes (e.g., trisomy 21), and metabolic conditions (Osborne et al., 2010), reviewed in Paciorkowski et al. (2011a).

LGS, which typically has onset between 1 and 8 years, is characterized by mixed seizure types—including tonic, atonic, myoclonic, atypical absence, focal, and generalized tonic–clonic seizures—and intellectual disability (Camfield, 2011). The characteristic electroencephalography (EEG) patterns include a slow (<2.5 Hz) spike-wave pattern and generalized paroxysmal fast activity. Patients with IS and other early onset epilepsies may evolve into LGS, underscoring a likely shared etiology. As with IS, LGS may be symptomatic of underlying structural or metabolic abnormalities, but in about 25–35% of the cases, the cause remains unknown (Camfield, 2011).

In the past few years, de novo or inherited mutations in genes such as *ARX*, *STXBP1*, *CDKL5*, and *SCN1A* have been found in subjects with IS, although these genes seem to explain only a small percentage of the IS cases of hitherto unknown cause. Pathogenic CNVs have also been reported in children with IS (Mefford et al., 2011; Paciorkowski et al., 2011b). So far, there are far fewer established genetic etiologies for LGS. Since patients may evolve from one electroclinical syndrome to another over the course of childhood, and the genetic etiologies of the epileptic encephalopathies without known cause may be shared across syndromes, a study of both IS and LGS is likely to increase the chances for etiologic discovery. Given the relative severity of these disorders, the main hypothesis of project 1 is that a significant number of IS and LGS cases of unknown cause are due to dominant de novo mutations.

To undertake our gene discovery effort, we will conduct whole exome sequencing on 300 + family trios comprising patients with cryptogenic IS or LGS and their parents who do not have epilepsy (optimizing overlapping genetics by first selecting individuals who show evolution from IS to LGS). The probands and parents to be studied have been collected by the Epilepsy Phenome/Genome Project (EPGP) (see: http://www.epgp.org) and thus have detailed phenotypic data.

**Figure 2:**
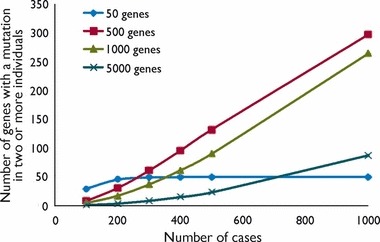
Modeling the number of cases required to identify causal genes, assuming various total numbers of genes involved in the disease. x-axis: number of cases included in the analysis; y-axis: expected number of genes with a mutation in two or more individuals. The model assumes each gene is equally likely to have a mutation, and calculations are based on a simple binomial distribution.

With the whole exome data we will screen for genes that have both de novo point mutations and de novo CNVs. We will also consider more complex genetics including genes with a combination of de novo and rare inherited mutations or genes with only rare inherited mutations. A recent study suggests that each new diploid genome has an estimated 70 de novo mutations and that only a small fraction of these are likely to fall in coding regions of the genome (∼1.5%) (Roach et al., 2010). To obtain a rough idea of whether our sample size is sufficient for this approach, we can assume that IS and LGS cases are due to a mutation in any one of N genes, and that each of the N genes is equally likely to be responsible for any given case. We can then estimate the number of genes expected to carry a causal mutation in more than one case as a function of N (Fig. 2). This analysis suggests that if the number of genes is <500 we will be well powered to identify some of the responsible genes with our study design. Finally, although the primary discovery strategy for the IS/LGS cohort will focus on identifying de novo mutations affecting the same genes, we will evaluate rare inherited variants of interest in larger case control comparisons (described below), and we will sequence genes of interest in additional cases and controls.

## Epi4K Project 2: Multiplex Families and Pairs

Project 2 of the Epi4K Center without Walls will use whole genome sequencing (WGS) to identify genomic variation that influences risk for common forms of IGE and NFE found in families with two or more affected members. The families to be studied include: (1) 1,500 pairs of first-degree relatives (sibling pairs or parent-offspring pairs) with either IGE or NFE collected in EPGP; and (2) approximately 300 families containing ≥3 individuals with IGE or NFE previously collected and phenotyped in detail by collaborating investigators. Our rationale for studying familial cases is that these cohorts are likely to be enriched for genetic influences on epilepsy, thereby greatly enhancing the likelihood of identifying the genes involved (Risch, 2001). This large, well-characterized dataset of relative pairs and multiplex epilepsy families provides an unparalleled opportunity for genetic analysis, both to detect clinically defined syndromes that have not previously been recognized, and to identify genomic variants that raise susceptibility to these specific syndromes.

One of the most challenging problems for genetic studies of the epilepsies is the extreme clinical and genetic heterogeneity of the disorder. Broadly defined by recurrent unprovoked seizures, the epilepsies encompass a wide range of different syndromes presumed to have different pathogenic mechanisms. Project 2 will therefore begin with an in-depth analysis of epilepsy phenotypes in the families to be studied. We will test the hypothesis that specific constellations of clinical epilepsy features aggregate within families and constitute “familial syndromes” that can be used to separate families into distinct subgroups likely to differ in their genetic influences.

We will then perform a comprehensive analysis of genomic variation in the families, including coding and noncoding single nucleotide variants, indels, and CNVs. In each family containing ≥3 affected individuals, we will sequence two affected individuals, selected to be as distantly related as possible. The rationale for this approach is that affected individuals within these multiplex families are likely to share the genetic variant(s) leading to their epilepsy, and distant relatives are unlikely to share as many variants by chance, so that examination of variants shared by the two distantly related affected individuals can be used as a filter for variants likely to have a pathogenic effect. To further enhance filtering, we also will genotype all affected family members for a panel of linkage markers and use the resulting information to examine whether the potentially pathogenic variants identified by sequencing are localized to genomic regions shared by all affected individuals in the family. In the EPGP first-degree relative pairs, we will perform a similar comprehensive analysis of genomic variation that influences risk for either IGE or NFE. WGS will be carried out in one individual from each of the 1,500 relative pairs (genome sequencing the second individual would provide little additional information, since the relatives are so closely related, but the second individual will be valuable for testing for the presence of proposed pathogenic variants). To improve filtering, we will also genotype the EPGP full siblings for a linkage panel of markers and examine whether regions containing potentially pathogenic variants in the sequenced sibling are also inherited in the other sibling.

While control samples will not be sequenced directly through Epi4K, all of our association analyses will make use of the rapidly growing number of genomes that have been sequenced from a variety of sources. In particular, we anticipate having available several thousand genomes (a combination of exomes and genomes) sequenced at Duke University, and additional thousands available through public databases. This will allow us to implement both variant-based and gene-based association tests comparing the epilepsy genomes to population-control genomes.

We expect that numerous potentially disease-related variants will be identified; their likelihood of being pathogenic will be ranked by the extent of cosegregation with epilepsy within larger families, their recurrence in other families or relative pairs across the whole dataset and their predicted functional effects. Once our initial association analyses and filtering analyses have been performed, further validation of selected variants and genes of interest will be done by follow-up association studies in independent samples and functional genomic studies, both within Epi4K and with additional collaborators. We anticipate having available as many as 10,000 further samples for this effort, combining both further epilepsy samples and control samples. These samples will be drawn primarily from the EPIGEN consortium and from control samples available at Duke University and the University of Washington. Finally, we will assess the specificity of effect of identified variants for different clinically defined subsets of epilepsy and for families containing different numbers of affected individuals. In these analyses, we hypothesize that the variants that influence risk will differ between IGE and NFE, and among the syndromes identified in our analysis of familial phenotypes.

## Epi4K Project 3: Prognosis

The epilepsies have a highly variable prognosis, even among persons with seemingly the same type of epilepsy. Perhaps the most important aspect of epilepsy prognosis is how well a patient responds to medication, with the prevalence of pharmacoresistance in epilepsy largely unchanged by the new antiepileptic drugs (AEDs).

Project 3, which is planned as a future activity within Epi4K, will use established epilepsy cohorts with well-characterized data on seizure outcome to systematically explore the relationship between genetic variation and prognosis, focusing in particular on pharmacologic control of seizures. Our overarching hypothesis is that genetic variation influences the likelihood of response to specific AEDs. While the study design and discovery paradigms are agnostic about whether the variants that are most relevant to prognosis also influence disease predisposition, we postulate a genetically based interrelatedness of disease etiology and outcome (i.e., specific susceptibility variants increase the risk of subtypes of epilepsy that are inherently less responsive to currently available AEDs) (Johnson et al., 2003; Rogawski & Johnson, 2008).

The clinical data to be used in project 3 have been collected within established pharmacogenetic studies of epilepsy from the United Kingdom, United States, and Australia, providing a large combined cohort of newly treated epilepsy with prospectively measured seizure and drug response outcomes (see Table 1). The prospective epidemiologic design offers many advantages for the study of drug response outcomes including the facility to record uniform and unbiased patient data over time (Leschziner et al., 2006; Manolio et al., 2006). Prospective studies also mitigate many of the problematic aspects of the retrospective case–control design, such as choice of controls, recall bias, varying definitions of AED response, and potential biases in the allocation of outcome status. However, the inherent high-cost and long-term nature of prospective studies restricts the accrual of large numbers of cases at the extremes of phenotypic variation. Therefore, we will capitalize on the strengths of the collaboration to provide large numbers of appropriately phenotyped cases and include, in addition to the prospective study, a matched case–control (retrospective) design focusing on patients at the extremes of the drug-response phenotype (i.e., extreme responders and extreme treatment resistance).

Our approach to the genetic analysis of these cohorts is twofold. First, we will determine the prognostic implications of all variants identified as risk factors for epilepsy by genotyping susceptibility variants identified in Epi4K across the whole prospective epilepsy cohort (n = 2,000). In this way, Epi4K will be able to leverage its established clinical resources not only for discovering susceptibility variants, but also for exploration of the prognostic implications of susceptibility variants. Second, we aim to exome-sequence patients at the extremes of drug-response phenotype, as well as across the prospective cohort, to identify genetic variants influencing response to medication independent of disease susceptibility.

## Epi4K Project 4: Copy Number Variants

**Figure 3:**
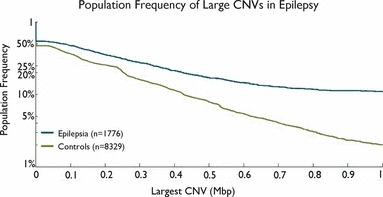
CNV size in patients with epilepsy (blue line) compared to population controls (green). Adapted from Cooper et al. (2011)

CNVs play an important role in the genetic risk and etiology of epilepsy. Large, recurrent CNVs as well as rare, nonrecurrent CNVs have now been identified in a proportion of patients with generalized, focal, or encephalopathic epilepsies. The burden for large CNVs (5–10%) is significant when compared to controls (Fig. 3) and is comparable to those reported for children with autism (Cooper et al., 2011), albeit less so than for children with developmental delay and congenital abnormalities. Current platforms for detecting genome-wide CNV include array comparative genomic hybridization (CGH) and single nucleotide polymorphism (SNP) genotyping platforms. Although both have been used with great success to identify large CNVs, the ability to routinely and robustly detect CNVs <50 kb by either of these platforms is limited and largely restricted to unique regions of the genome (Alkan et al., 2011a). As a result, the role of small- to moderate-sized CNVs in disease is largely unknown and we predict will contribute significantly to the etiology of epilepsy. With the development of next generation sequencing, we now have a unique opportunity to detect the size spectrum of CNVs more comprehensively—from a single base pair to many megabases. Early studies confirm the ability to detect CNVs that are not detected by other platforms, increasing the rate of CNV discovery by more than fivefold (Hormozdiari et al., 2009; Conrad et al., 2010; Pelak et al., 2010; Mills et al., 2011). With the continued development and improvement of novel algorithms (Karakoc et al., 2011), the detection of CNVs from next-generation sequence data has improved significantly, but more work needs to be done to capture more complex variation as well as specific classes such as inversions and novel insertions. Data from Epi4K, which will include exome and genome sequences of 4,000 patients with various types of epilepsy, will be an incredibly rich resource for identifying disease-associated CNVs that are important within and across epilepsy subtypes.

The primary goal of Epi4K project 4 will be to apply novel computational algorithms to whole-exome and whole-genome data for Epi4K patients to detect epilepsy-associated CNVs. Our approach will be to focus on structural variation in protein-coding sequence of all 4,000 individuals using a combination of algorithms based on read-depth and split-read analyses. Currently, there are three basic strategies that may be applied to the detection of structural variation from sequence data: read-pair, read-depth, and split-read methods (Medvedev et al., 2010; Alkan et al., 2011b; Mills et al., 2011). Read-pair methods detect structural variation based on discordances in length and/or orientation and can, in principle, detect insertions, deletions, duplications, and inversions. Read-depth methods detect regions of significant excess or depletion of sequence reads as surrogates of duplications and deletions, respectively. Recently developed methods taking advantage of algorithms that map to multiple locations allow copy-number variation of genes within nonunique regions to be accurately assayed (Sudmant et al., 2010). Here, genome coverage is a key aspect in the power to detect events as a function of size (Bailey et al., 2002; Alkan et al., 2009; Yoon et al., 2009). Split-read methods detect breakpoints based on a read spanning a breakpoint and, as a result, length of the sequence read as well as the sequence complexity of the breakpoint determine sensitivity (Karakoc et al., 2011). In general, maximal discovery benefits from application of read-pair, read-depth, and split-read methods on the same datasets, and we will develop appropriate pipelines for the evaluation of exome data and whole genome data and for comparison of the set of CNVs identified in each set. Potentially pathogenic CNVs and structural variants will be subjected to experimental validation and familial segregation analysis, where applicable, and to further follow-up.

## Conclusions

Epi4K builds upon the substantial work that has already been focused on understanding the genetic basis of common forms of epilepsy and epileptic encephalopathies, including the prognostic determinants of these disorders. Our approach emphasizes careful phenotyping of subjects, the increased accessibility of next-generation sequencing, state-of-the-art methods for genomic analysis, and the unique opportunities that are created by large-scale collaborations. If successful, this work will lead to advances in our ability to identify the causes of many forms of so-called idiopathic and cryptogenic epilepsy, and it will drive the development of drugs that are antiseizure, and perhaps (by focusing on the interaction between etiology and outcome) even disease-modifying or antiepileptogenic. Of course, the identification of genetic risk factors for epilepsy is only a first step in such efforts, and new research programs building on a variety of platforms (e.g., knockin and knockout mouse models, high-throughput small molecule screens, in silico cellular and network analyses), will be required to study the biologic bases of those genetic risks and to assess their translational implications. To this end, the Epi4K investigators welcome the involvement of other researchers from throughout the world who see a potential benefit in collaborating with us. Those interested are encouraged to contact us via our website (http://www.epgp.org/epi4K).
